# A foliar pigment-based bioassay for interrogating chloroplast signalling revealed that carotenoid isomerisation regulates chlorophyll abundance

**DOI:** 10.1186/s13007-022-00847-5

**Published:** 2022-02-17

**Authors:** N. Dhami, B. J. Pogson, D. T. Tissue, C. I. Cazzonelli

**Affiliations:** 1 Hawkesbury Institute for the Environment, Western Sydney University, Locked Bag 1797, Penrith, NSW 2751 Australia; 2 Australian Research Council Centre of Excellence in Plant Energy Biology, Research School of Biology, The Australian National University, Canberra, ACT 2601 Australia; 3 School of Health and Allied Sciences, Pokhara University, Pokhara 30, Kaski, Gandaki 33700 Nepal

**Keywords:** Carotenoid isomerisation, Chlorophyll, Chloroplast, Retrograde signal, Plastid development, Apocarotenoid, *Arabidopsis*

## Abstract

**Background:**

Some plastid-derived metabolites can control nuclear gene expression, chloroplast biogenesis, and chlorophyll biosynthesis. For example, norflurazon (NFZ) induced inhibition of carotenoid biosynthesis in leaves elicits a protoporphyrin IX (Mg-ProtoIX) retrograde signal that controls chlorophyll biosynthesis and chloroplast development. Carotenoid cleavage products, known as apocarotenoids, also regulate plastid development. The key steps in carotenoid biosynthesis or catabolism that can regulate chlorophyll biosynthesis in leaf tissues remain unclear. Here, we established a foliar pigment-based bioassay using *Arabidopsis* rosette leaves to investigate plastid signalling processes in young expanding leaves comprising rapidly dividing and expanding cells containing active chloroplast biogenesis.

**Results:**

We demonstrate that environmental treatments (extended darkness and cold exposure) as well as chemical (norflurazon; NFZ) inhibition of carotenoid biosynthesis, reduce chlorophyll levels in young, but not older leaves of *Arabidopsis*. Mutants with disrupted xanthophyll accumulation, apocarotenoid phytohormone biosynthesis (abscisic acid and strigolactone), or enzymatic carotenoid cleavage, did not alter chlorophyll levels in young or old leaves. However, perturbations in acyclic *cis*-carotene biosynthesis revealed that disruption of CAROTENOID ISOMERASE (CRTISO), but not ZETA-CAROTENE ISOMERASE (Z-ISO) activity, reduced chlorophyll levels in young leaves of *Arabidopsis* plants. NFZ-induced inhibition of PHYTOENE DESATURASE (PDS) activity caused higher phytoene accumulation in younger *crtiso* leaves compared to WT indicating a continued substrate supply from the methylerythritol 4-phosphate (MEP) pathway.

**Conclusion:**

The *Arabidopsis* foliar pigment-based bioassay can be used to differentiate signalling events elicited by environmental change, chemical treatment, and/or genetic perturbation, and determine how they control chloroplast biogenesis and chlorophyll biosynthesis. Genetic perturbations that impaired xanthophyll biosynthesis and/or carotenoid catabolism did not affect chlorophyll biosynthesis. The lack of CAROTENOID ISOMERISATION reduced chlorophyll accumulation, but not phytoene biosynthesis in young leaves of *Arabidopsis* plants growing under a long photoperiod. Findings generated using the newly customised foliar pigment-based bioassay implicate that carotenoid isomerase activity and NFZ-induced inhibition of PDS activity elicit different signalling pathways to control chlorophyll homeostasis in young leaves of *Arabidopsis*.

## Introduction

The level of photosynthetic pigments (chlorophylls and carotenoids) in leaves is tightly coordinated with chloroplast development and can change during development or in response to environmental stress. Older leaves from *Arabidopsis* contain enlarged chloroplasts that can sustain the steady-state turnover of pigments [7, 8, 34]. However, the level of chlorophylls and carotenoids is approximately 40% higher in younger leaves comparison to older leaves of *Arabidopsis* [23]. Younger *Arabidopsis* leaves, in comparison to older leaves, harbour actively dividing and expanding cells, leading to a net increase in total cell number and chloroplast capacity per area to store pigments for photosynthesis [21, 30]. This observation correlates with higher rates of photosynthesis in recently emerged leaves compared to mature leaves of *Arabidopsis* [65]. The younger leaves of *Arabidopsis* are more tolerant to the excessive light exposure compared to mature leaves [11, 20], and unlike older leaves they can modify their pigment levels upon exposure to elevated CO_2_ [23]. Developing chloroplasts within the actively dividing cells in younger leaves can alter their biogenesis in response to environmental change, while older leaves that contain fully expanded cells with mature chloroplasts are turned over slowly [21]. This plasticity intrinsic to young *Arabidopsis* leaves could be utilised to develop an *in planta* biossay to decipher how environmental, chemical, and/or genetic perturbations regulate chloroplast biogenesis and development.

**Fig. 1:**
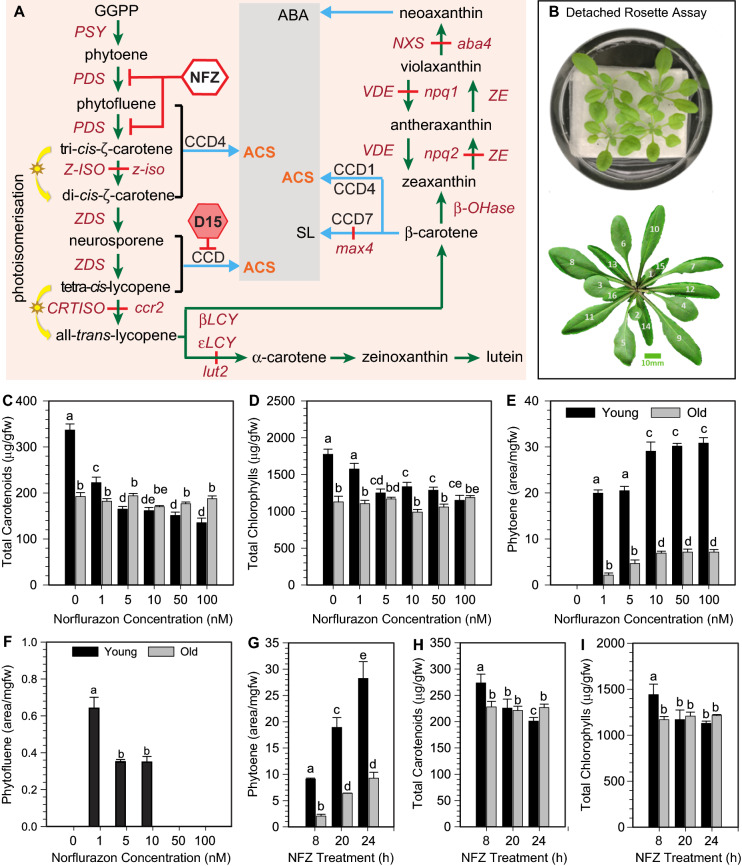
Optimisation of a pigment-based signalling bioassay in *Arabidopsis* detached rosettes. **A** Pathway for carotenoid biosynthesis and catabolism into an apocarotenoid signal (ACS) or phytohormone such as strigolactone (SL) and abscisic acid (ABA). Norflurazon (NFZ) inhibits PDS activity and aryl-C3N hydroxamic acid (D15) impairs CCD activity. Green arrows and red lines represent positive and negative regulation, respectively. Blue lines denote a pathway towards the generation of a carotenoid cleavage product specified in the grey box. Mutants used in this study include; *ζ*-*carotene isomerase* (*z-iso*) and *carotenoid chloroplast regulatory 2* (*ccr2*), *lutein deficient 2* (*lut2*), *nonphotochemical quenching 1* (*npq1*), *nonphotochemical quenching 2* (*npq2*), *abscisic acid deficient 4* (*aba4*), *more axillary branching 3* (*max3*)*.* Abbreviations: GERANYLGERANYL PYROPHOSPHATE (GGPP), PHYTOENE SYNTHASE (PSY), PHYTOENE DESATURASE (PDS), ζ-CAROTENE DESATURASE (ZDS), ZETA-CAROTENE ISOMERASE (Z-ISO), CAROTENOID ISOMERASE (CRTISO), LYCOPENE EPSILON CYCLASE (LCY), and LYCOPENE BETA CYCLASE (bLCY), ZEAXANTHIN EPOXIDASE (ZE), VIOLAXANTHIN DEEPOXIDASE (VDE), NEOAXANTHIN SYNTHASE (NXS), CAROTENOID CLEAVAGE DIOXYGENASE (CCD). **B** Visual display of the whole rosette bioassay showing the rosettes incubating on kim wipes saturated with NFZ contained with a petri dish. The numbered rosette shows the leaf position by chronological age (1 to 15; oldest to youngest). Three week old *Arabidopsis* rosettes were treated with different NFZ concentrations (0–100 μM) for 24 h (**D**–**F**) and various time points over a 24 h period (**G**–**I**). *Arabidopsis* trays were kept in dark for 4–5 h before transferring *Arabidopsis* plants to NFZ under continuous light (130–150 µmol m^−2^ s^−1^, cool fluorescent lamps) at 22 °C. Mature (leaf 1–2; old) and recently emerged (leaf 9–11, young) leaves were collected after treatments and pigment levels quantified. Absolute concentrations of Phytofluene (**C**), Phytoene (**D**), Total carotenoids (**E**), and Total chlorophylls (**F**) in response to the different concentration of NFZ. Absolute concentrations of Phytoene (**G**), Total carotenoids (**H**), and Total chlorophylls (**I**) in response to the different NFZ incubation times over a 24 h period. Plots represent the mean values with standard error of means (n = 3–4; **C**) from a representative dataset of at least two independent experimental repetitions. Letter codes in the plots indicate the level of statistical variation (p < 0.05) in carotenoid content within and across the test groups determined by One-Way ANOVA adopting Holm-Sidak *post-hoc* multiple comparisons

In leaves, chloroplasts develop from either the etioplast or proplastid [15, 62]. Photosynthetic complexes in the chloroplast thylakoid antennae require the assembly of chlorophylls, carotenoids (lutein, β-carotene, violaxanthin, and neoxanthin), nucleus-encoded protein subunits, and redox-active co-factors (e.g. hemes and iron–sulfur clusters) to contribute to electron transfer reactions during photosynthesis as well as facilitate light harvesting and photo-protection [6]. Blocking carotenoid biosynthesis in foliar tissues impacts plastid development by disrupting thylakoid formation, triggers changes in photosynthesis-associated nuclear gene expression [49]. Norflurazon (NFZ) is commonly used to inhibit PHYTOENE DESATURASE (PDS) activity and block downstream carotenoid accumulation (Fig. 1A). The supply of substrates from the methylerythritol 4-phosphate (MEP) pathway continue to facilitate phytoene biosynthesis in *Arabidopsis* leaves, etiolated seedling and/or shoot derived calli, despite the NFZ-induced impairment in plastid development [50, 58, 63, 64]. The NFZ-treated tissues accumulate chlorophyll biosynthesis intermediate metabolites (e.g. protoporphyrin IX; Mg-ProtoIX) that act as retrograde signals to downregulate photosynthesis-associated nuclear gene expression in leaves [37]. Retrograde metabolites generated by the chloroplast provide “biogenic control” during early chloroplast differentiation from proplastids or etioplasts in emerging leaves, and/or “operational control” in mature leaf chloroplasts in response to environmental stimuli [52, 71]. What remains unknown is if NFZ also perturbs the biosynthesis of a downstream carotenoid-derived signal that regulates chloroplast biogenesis in young leaves.

Catabolism of carotenoids is a continuous process in leaves occurring via enzymatic and non-enzymatic oxidative cleavage [7, 8, 63]. Carotenoids can be cleaved enzymatically by CAROTENOID CLEAVAGE DIOXYGENASE (CCD) and 9-*CIS*-EPOXYCAROTENOID DIOXYGENASE (NCED) to generate apocarotenoids such as strigolactone (SL) and abscisic acid (ABA) phytohormones, respectively (Havaux, 2014, [56, 63]. SL and ABA control developmental and physiological processes such as shoot bud outgrowth and stomatal closure respectively, yet a function in modulating chloroplast development remains less clear [27, 48]. Exogenous application of apocarotenoids such as β-cyclocitral, anchorene, loliolide, β-cyclogeranic acid, and β-ionone have been shown to regulate nuclear gene expression, pigment accumulation in plastids, and/or stress acclimation responses in plant tissues [27, 48]. Some β-carotene-derived apocarotenoids require enzymatic cleavage by CCD1 or CCD4, mutants of which accumulate β-carotene in seeds and/or senescing leaves [4, 31, 59]. Zeaxanthin can be cleaved by a CCD subfamily member to generate zaxinone in rice that regulates growth [68], or oxidatively cleaved into apocarotenoids that exert an ABA-independent regulation upon gene expression [41]. What remains unknown is if mutants that perturb xanthophyll biosynthesis, or CCD mediated carotenoid catabolism, can alter chlorophyll levels in young and/or old leaf types.

An unidentified apocarotenoid signal (ACS) generated during acyclic *cis*-carotene biosynthesis has been shown to regulate nuclear gene expression and chloroplast biogenesis in *Arabidopsis* tissues [5, 14, 26]. The loss of ZETA-CAROTENE DESATURATE (ZDS) function causes lethality following photomorphogenesis, yet the albino seedlings accumulate *cis*-carotenes that were linked to the control of plastid development and formation of needle-like leaf phenotype [5, 24, 26]. The loss-of-function in ZETA-CAROTENE ISOMERASE (Z-ISO) causes *cis*-carotenes to accumulate in etiolated tissues and can delay chlorophyll biosynthesis during seedling photomorphogenesis [9, 18]. Also, the loss-of-function in CAROTENOID ISOMERASE (CRTISO) activity in *Arabidopsis* causes *cis*-carotene accumulation during seedling skotomorphogenesis, as well as in newly emerged leaves from plants grown under a shorter photoperiod, and triggers to accumulate an unknown *cis*-ACS that regulates plastid development and photosynthetic nuclear gene expression in a retrograde-like manner [14, 50]. Light-mediated photoisomerization of *cis*-carotenes compensates for the lack of isomerase activity in foliar tissues, presumably by reducing substrate availability required to make *cis*-ACS. What remains untested is if a *cis*-ACS can be generated under longer photoperiods to regulate chlorophyll levels accordingly in leaf-age specific manner.

In this paper, we demonstrate that extended darkness, cold exposure, and NFZ treatment reduce chlorophyll and carotenoid accumulations in young, but not old leaves of *Arabidopsis*. An *Arabidopsis* foliar pigment-based signalling bioassay was established to decipher key steps in carotenoid biosynthesis and/or catabolism that might generate an apocaroteoid signal controlling chloroplast development and chlorophyll levels in younger leaves. Genetic and chemical inhibitors of carotenogenesis were used to differentiate between the effects of carotenoid- and chlorophyll-derived signals respectively. We assume a change in chlorophyll levels in younger leaves could reflect a change in chloroplast biogenesis. We reveal new insights into how carotenoid isomerisation is the key rate-limiting step in the pathway mediating production of a signal that controls chlorophyll accumulation in the younger foliar tissues of *Arabidopsis* grown under a long photoperiod.

## Results

### Norflurazon inhibition of PDS activity reduces chlorophyll levels in young expanding leaves

An *in-planta* pigment-based signalling bioassay was developed using detached whole rosettes from *Arabidopsis* treated with different concentrations and durations of NFZ that inhibits carotenoid biosynthesis (Fig. 1A, B). Under control growth conditions, chlorophyll and carotenoid levels were significantly higher in younger (leaves 9 to 13) compared to old (leaves 1 to 4) leaf types (Fig. 1B–D). All NFZ concentrations (1 μM to 100 μM) caused phytoene to accumulate in both young and old leaf types, yet detectable levels of phytofluene were only apparent in young leaves at lower concentrations (1 and 10 μM) (Fig. 1E, F). At lower NFZ concentrations (1 µM), young leaves showed a reduction in total carotenoids, but not total chlorophylls (Fig. 1C, D). Total chlorophyll and carotenoid levels were significantly reduced in young leaves exposed to 5, 10, 50 and 100 μM of NFZ, yet their levels remained almost unchanged in older leaves. The absence of phytofluene and reduced chlorophyll levels in younger leaves exposed to 50 μM NFZ indicated this concentration was best suitable to further optimise the duration of NFZ treatement.

The impact of three durations (8, 20, 24 h) of NFZ treatment (50 μM) on pigment levels were assessed in young and old leaves. Phytoene levels were two- to threefold higher in young relative to old leaves after 8, 20 and 24 h of NFZ treatment (Fig. 1G). Detectable levels of phytoene could be observed within 4 h of NFZ treatment in younger leaves (data not shown). After 8 h of NFZ treatment, the total chlorophyll and carotenoid levels remained higher in younger leaves, whereas a significant reduction was observed in young leaves after 20–24 h (Fig. 1H, I). Therefore, 24 h of treatment with 50 uM NF shows a clear reduction in chlorophylls in young, but not old mature leaves, thereby providing a *in planta* pigment-based bioassay to decipher which environmental factors and what rate-limiting steps in carotenogenesis impact plastid development.

### Exposure of young leaves to cold and darkness reduces pigment levels

**Fig. 2:**
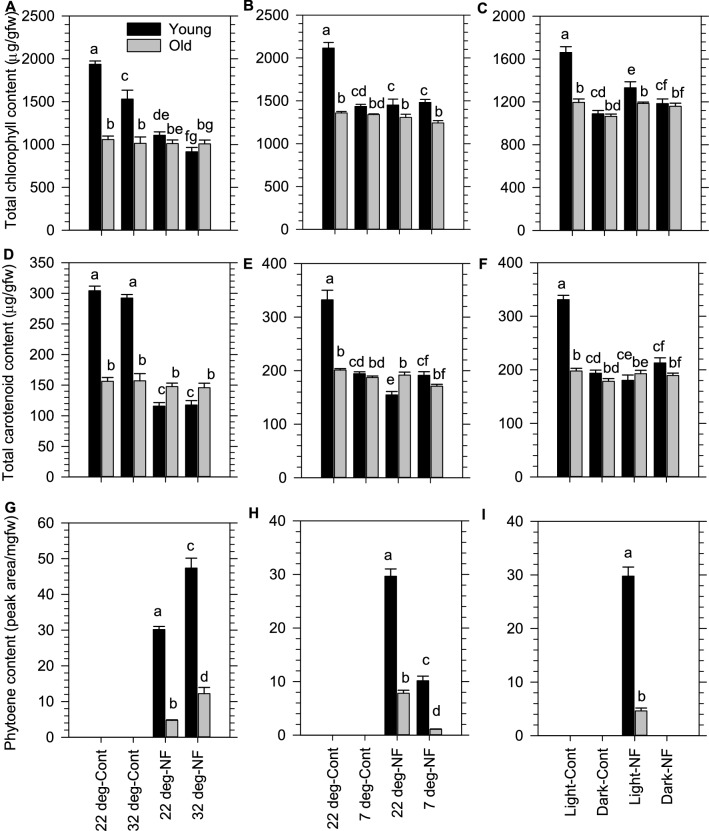
Chlorophyll and carotenoid content in *Arabidopsis* rosette leaves exposed to changes in temperature and extended darkness with or without norflurazon. Total chlorophyll (**A**–**C**), Total carotenoid (**D**–**F**), and Phytoene **(G**–**I**) content in young and old leaves from the *Arabidopsis* rosettes exposed to warm (32 °C; **A**, **D**, **G**), cold (7 °C; **B**, **E**, **H**), and darkness (**C**, **F**, **I**) in the presence or absence of NFZ. Error bars display standard error of the mean (n = 4). Dataset is representative of two independent experiments. Letter codes denote statistical variation (p < 0.05) determined by Two-Way ANOVA with Holm-Sidak *post-hoc* multiple comparison

The impact of warm (32 °C) and cold (7 °C) temperatures, and extended darkness (24 h) on plastid developed was examined using the pigment-based signalling bioassay. None of these treatments affected chlorophyll or carotenoid levels in older mature leaves (Fig. 2A–F). Similarly, these treatments did not cause phytoene to accumulate in any leaf types in the absence of NFZ (Fig. 2G–I). In contrast, young leaves exposed to the cold and darkness showed reduced chlorophyll and carotenoid levels that became equivalent to that of old leaves (Fig. 2B, C, E, F), whereas warmer temperature slightly decreased chlorophyll levels and had no significant impact on carotenoid content (Fig. 2A, D). Therefore, young leaves were highly amenable to alter their pigment levels in response to cold and darkness, while old leaves remained resilient to any environmental change.

The impact of environmental change on pigment levels in aging leaf types was further investigated in combination with NFZ. Both young and old leaves from the NFZ-treated plants accumulated phytoene under warmer and colder conditions, but phytoene did not accumulate in dark exposed leaves (Fig. 2G–I). The levels of phytoene were consistently higher in young compared to older leaves. Compared to the standard 22 °C growth temperature, warmer and colder temperatures significantly enhanced and reduced phytoene accumulation respectively, in both leaf types (Fig. 2G–I). Intriguingly, none of the dark-exposed leaves accumulated any phytoene during NFZ treatment (Fig. 2I). Compared to their respective control without NFZ, total chlorophylls and carotenoids were significantly reduced in young, but not old leaves from plants treated with NFZ and exposed to 32 °C, 7 °C, and darkness (Fig. 2A–F). The trends with or without NFZ were rather similar. Overall, NFZ in combination with warm, cold or dark treatments does not create an obvious additive change on total chlorophyll or carotenoid levels in either leaf-type, despite higher, lower and absent levels of phytoene in NFZ-treated leaf tissues exposed to warm, cold and dark treatments respectively.

### Perturbing strigolactone, abscisic acid or xanthophyll biosynthesis does not alter chlorophyll content in young leaves

**Fig. 3:**
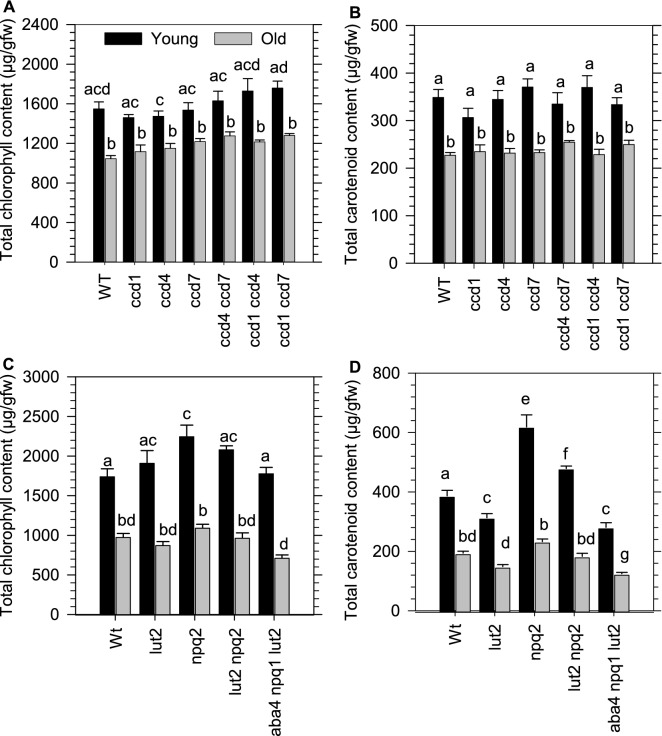
Chlorophyll and carotenoid content in young and old leaves from *carotenoid cleavage dioxygenase* (*ccd*), xanthophyll, SL and ABA mutants. The average total chlorophyll (**A**, **C**), and carotenoid (**B**, **D**) content in young and old leaves from WT and mutants are displayed with error bars denoting the standard error (n = 4). Data is representative of two experimental repetitions. Letters denote statistical variation (p < 0.05) within leaf types across different germplasm determined using a Two-Way ANOVA and *post-hoc* Holm-Sidak multiple comparison. *ccd1; carotenoid cleavage dioxygenase, ccd4; carotenoid cleavage dioxygenase 4, ccd7; carotenoid cleavage dioxygenase 7 (max3), ccd8; carotenoid cleavage dioxygenase 8 (max4); npq1*; *nonphotochemical quenching 1 (violaxanthin deepoxidase), npq2*; *nonphotochemical quenching 2 (zeaxanthin deepoxidase), aba4*; *abscisic acid deficient 4 (neoxanthin synthaseS), lut2*; *lutein defecient 2 (epsilon-lycopene cyclase)*

We investigated if blocking SL, ABA, and xanthophyll biosynthesis could impact chlorophyll biosynthesis in young leaves. Total chlorophyll and carotenoid levels in young leaves remained high relative to old leaves in the loss-of-function in single (*ccd1*, *ccd4*, *ccd7*) or double (*ccd1 ccd4*, *ccd7 ccd 4*, *ccd 1 ccd 7*) mutants that impaired CAROTENOID CLEAVAGE DIOXYGENASE (CCD) activity (Fig. 3A, B). Therefore, it appears unlikely that SL generated from CCD7 cleavage, or an ACS produced from cleavage by CCD1 and/or CCD4, regulates chlorophyll levels in young leaves.

Mutants that block the production of lutein (*lut2*), violaxanthin and neoxanthin (*npq2*), lutein, violaxanthin and neoxanthin (*npq2 lut2*), lutein and neoxanthin (*aba4 npq1 lut2*), or hyperaccumulate zeaxanthin (*npq2, npq2 lut2*), did not affect the higher chlorophyll levels in younger relative to older leaves (Fig. 3C, D). There were differences in the total carotenoid content among the different mutation combinations, however, it was always higher in young relative to older leaves mirroring the same trend observed in chlorophyll levels. Therefore, perturbations in xanthophylls that are required for canonical ABA biosynthesis does not appear to affect chlorophyll accumulation in young *Arabidopsis* leaves.

### Carotenoid isomerase activity regulates chlorophyll levels in young leaves

**Fig. 4:**
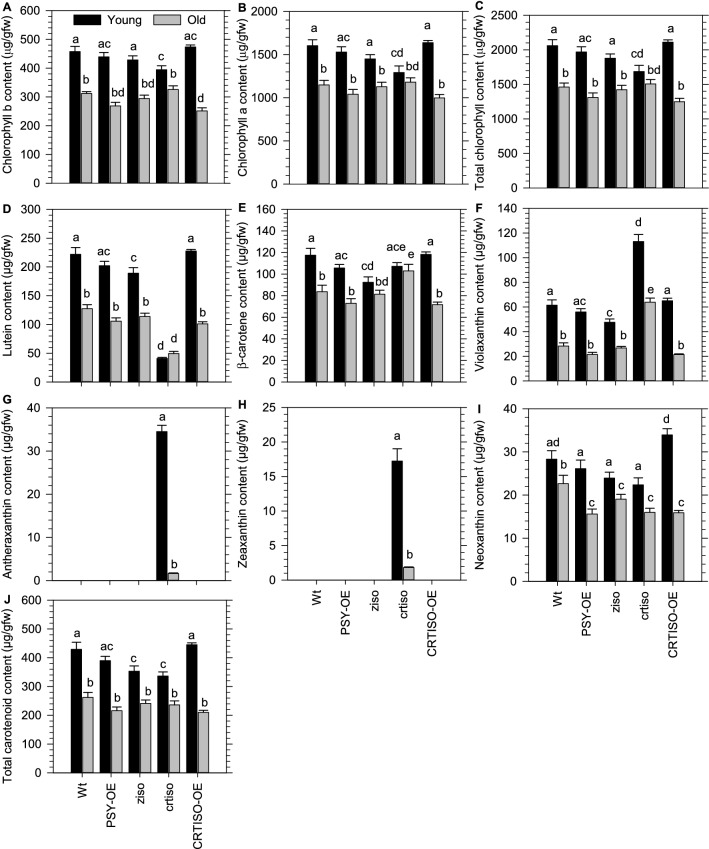
Chlorophyll and carotenoid content in young and old leaves of *Arabidopsis* germplasm that alter *cis*-carotene biosynthesis. **A** Chlorophyll b, **B** Chlorophyll a, **C** Total chlorophyll, **D** Lutein, **E** β-carotene, **F** Violaxanthin, **G** Neoxanthin, **H** Antheraxanthin, **I** Zeaxanthin, and **J** Total carotenoids were quantified in young and old leaves of different germplasm. Mean values are displayed with standard error (n = 4) being a representative dataset from two experimental repetitions. Letters denote statistical variation (p < 0.05) within leaf types across different germplasm determined using a Two-Way ANOVA and *post-hoc* Holm-Sidak multiple comparison. *ziso* and *crtiso* are loss of function mutants in *ζ-carotene isomerase* and *carotenoid isomerase*, respectively. *PSY-*OE enables higher PSY activity (35S::*AtPSY*#23) [46] and 35S::At*CRTISO* restored CRTISO activity to the loss-of-function *ccr2.1* mutant [16]

We investigated if the major rate-limiting step in carotenoid biosynthesis enabled by PSY could regulate pigment levels in leaves. Like WT, the content of individual, as well as total chlorophylls and carotenoids, were higher in young compared to old leaves from a transgenic line overexpressing *PSY* (35S::*AtPSY*#23) [46] (Fig. 4A–J). However, there were no major differences in pigment levels between *PSY*-OE and WT in the respective leaf types, with the exception for a subtle reduction in neoxanthin in older leaves from *PSY*-OE. Therefore, overexpression of *PSY* did not impact chlorophyll levels in young or old leaves.

Next, we investigated if the loss-of-function in *z-iso* or *crtiso* mutants, that accumulate acyclic *cis*-carotenes under light limiting conditions, could trigger a change in chlorophyll levels in young leaves from plants grown under long photoperiod. The chlorophyll content in *z-iso* young leaves was higher than old leaves (Fig. 4A–C). The level of lutein, β-carotene, violaxanthin, and, hence, total carotenoids was slightly lower in young leaves of *z-iso* compared to the young leaves from the WT. The carotenoid content in old leaves from both *z-iso* and WT were identical (Fig. 4D–J). The loss-of-function of *CRTISO* (*ccr2.1*) caused a reduction in total chlorophyll content in young leaves, such that it was similar to old leaves (Fig. 4C). The young leaves from *crtiso* showed lower *chlorophyll b* content compared to WT, whereas *chlorophyll a* content was severely reduced and identical to WT older leaves. The level of chlorophylls in older leaves of *crtiso* and WT were similar (Fig. 4A, B). Total carotenoid content was significantly lower in young leaves from *crtiso* relative to WT, yet carotenoid levels were similar in older leaves (Fig. 4J). Transgenic overexpression of CRTISO (35S::At*CRTISO* pMDC32:CRTISO: *CRTISO*-OE) in the *crtiso* mutant (*ccr2.1*) restored total chlorophyll and carotenoid levels in young leaves back to WT levels (Fig. 4C, J). Unlike WT, the level of lutein and β-carotene were similar in young and old leaves from *crtiso* (Fig. 4D, E). Whereas, vioaxanthin, antheraxanthin, zeaxanthin and neoaxanthin were all significantly higher in younger compared to older leaves from *crtiso*. Therefore, the reduction in chlorophyll in young but not older leaves of *crtiso*, reveals that photoisomerization of *cis*-carotenes cannot maintain the higher chlorophyll levels normally quantified in young WT leaves.

### NFZ and carotenoid isomerase activity regulate chlorophyll levels differently in young leaves

**Fig. 5:**
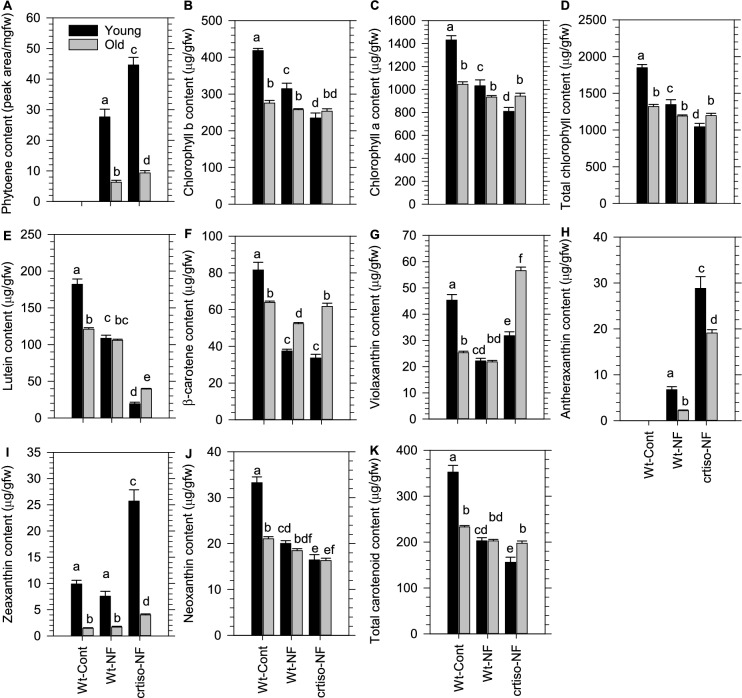
Norflurazon-induces additive changes in chlorophyll and carotenoid content in the *carotenoid isomerase* mutant leaves. **A** Phytoene, **B** Chlorophyll b, **C** Chlorophyll a, **D** Total chlorophyll, **E** Lutein, **F** β-carotene, **G** Violaxanthin, **H** Antheraxanthin, **I** Zeaxanthin, and **J** Neoxanthin, **K** Total carotenoids in young and old leaves from *crtiso* and WT plants exposed to norflurazon. Mean values are displayed with standard error (n = 4) being a representative dataset from two experimental repetitions. Letters denote statistical variation (p < 0.05) within leaf types across different germplasm determined using a Two-Way ANOVA and *post-hoc* Holm-Sidak multiple comparison. *crtiso*; *carotenoid isomerase*

We next assessed whether NFZ treatment and *crtiso* have synergistic effects on pigmentation in young leaves. NFZ-treatment further elevated phytoene levels in both young and old leaves of *crtiso* compared to WT (Fig. 5A). Curiously, phytoene content was significantly higher in young, relative to older leaves from *crtiso* and WT plants treated with NFZ revealing there is a continued isoprenoid supply for carotenoid biosynthesis. NFZ caused a reduction of chlorophylls in young leaves from WT, that was even more pronounced in young *crtiso* leaves displaying chlorophyll levels below that of older leaves (Fig. 5B–D). Similarly, total carotenoid content in young leaves from *crtiso* plants treated with NFZ were significantly lower than older leaves, while young leaves form NFZ treated WT plants showed carotenoid levels similar to older leaves (Fig. 5K). Hence, NFZ and *crtiso* might affect chlorophyll levels and perhaps chloroplast biogenesis by independent signalling pathways.

The impact of NFZ on individual carotenoid levels in young leaves from WT and *crtiso* treated with NFZ were assessed to determine how they impact the carotenoid biosynthetic pathway. NFZ reduced β-carotene levels in young leaves from both WT and *crtiso* to levels below that observed in older leaves (Fig. 5F). However, while NFZ reduced lutein levels in WT young leaves, it further reduced lutein content in the *crtiso* mutant to levels below that of older leaves, revealing an additive effect (Fig. 5E). Violaxanthin levels were reduced in young WT and *crtiso* leaves from plants treated with NFZ; even though the levels were substantially higher in older leaves from the *crtiso* mutant (Fig. 5G). NFZ treated younger leaves contained more antheraxanthin and zeaxanthin compared to older leaves, which was further elevated almost threefold in the *crtiso* mutant, evidence of continued carotenoid biosynthesis or reduced catabolism (Fig. 5H, I). The levels of neoxanthin were similar in young and older leaves from WT and *crtiso* plants treated with NFZ (Fig. 5J). The different impacts of NFZ on individual carotenoid levels in young leaves from WT and *crtiso* highlight ongoing and likely impaired chloroplast biogenesis, as well as continued isoprenoid substrate supply for carotenoid biosynthesis. The differential effects of NFZ treatment on accumulation of chlorophylls and carotenoids in *crtiso* relative to WT, reveal that they might signal different pathways to regulate chloroplast development.

## Discussion

The higher pigment content in young relative to old *Arabidopsis* leaves results from a greater cell and hence chloroplast density, that undergo rapid differentiation, division and expansion in emerging leaves providing them with plasticity to change in response to environmental, chemical and/or genetic perturbations [21, 23, 30, 34]. We demonstrate that extended darkness and cold exposure for 24 h can reduce chlorophyll by 20–50% to match levels as displayed by the more resilient older leaves. The optimised pigment-based signalling bioassay allowed detached *Arabidopsi*s rosettes to be exposed to chemicals such as NFZ, that in addition to inhibiting carotenoid biosynthesis, trigger a plastid derived signal that can impair plastid biogenesis and reduce chlorophyll levels in young, but not old leaf types. Mutations that disrupted xanthophyll biosynthesis and degradation into downstream phytohormones such as SL and ABA, as well as other apocarotenoids did not affect chlorophyll levels in young leaves. An unidentified acyclic *cis*-carotene derived ACS produced in tissues from *Arabidopsis* plants lacking function of the CAROTENOID ISOMERASE was recently shown to regulate chloroplast biogenesis in newly emerged leaves that manifested as a virescent phenotype in plants grown under a shorter photoperiod [14]. Here we demonstrate that *crtiso* mutant plants grown under a longer photoperiod have lower chlorophyll levels indicating that photoisomerisation can rate-limit the generation of a *cis*-ACS that perturbs plastid development. NFZ treatment of WT and *crtiso* mutant young leaves, revealed similar, opposite, as well as additive effects on individual pigment accumulations in young leaves. We propose that carotenoid isomerisation controls an unidentified *cis*-ACS that mediates a different signalling process to that elicited by NFZ (e.g. chlorophyllide or Mg-ProtoIX) in controlling chlorophyll biosynthesis and perhaps chloroplast development in young leaves of *Arabidopsis*.

### Norflurazon and environmental factors impede pigmentation in young emerging leaves

Our pigment-based signalling bioassay showed that NFZ caused a two- to threefold higher accumulation of phytoene in young compared to old leaves in agreeance with previous reports [8]. Despite a presumable impairment in plastid biogenesis in young leaves containing dividing cells and developing plastids, phytoene biosynthesis continued revealing a sufficient substrate availability from the MEP pathway. Inhibition of carotenoid biosynthesis by NFZ was previously shown to initially enhance pathway flux, presumably compensating for the short supply of β-carotene [8]. The fact that total pigment levels in old leaves following NFZ treatment were similar to the control revealed less plasticity and resilience in mature chloroplasts to maintain chlorophyll. The capacity for pigment accumulation in leaves varies by chloroplast developmental gradients along a given leaf axis (e.g. mature plastids at the tip in expanding cells, and differentiating plastids at the base of dividing cells), as well as between leaves of different ages (e.g. smaller/fewer plastids in young immature leaves undergoing cell division and expansion, and larger/numerous plastids in old mature leaves undergoing steady state turnover) [21, 30, 34]. There are proplastid to chloroplast transitions occurring within the shoot apical meristem (SAM) of the shoot apex, where flanking leaf primordia emerge as young leaves containing chloroplasts and leucoplasts with developing grana and thylakoids [17]. While mature chloroplasts can undergo a slow steady-state carotenoid turnover [7], this was not evident within our 24 h bioassay. Therefore, we attribute the NFZ-induced concurrent decrease in chlorophyll and carotenoid content in young leaves to a perturbation in chloroplast biogenesis in rapidly dividing and expanding cell types.

Extended darkness reduced total chlorophyll and carotenoid levels in young leaves mimicking the pattern exerted by NFZ. In *Arabidopsis*, carbon stored in the chloroplasts during the day as starch are remobilized during the night to support sugar metabolism, and excessive accumulation of sugars in the *maltose excess 1* mutant (*mex1*) cause chloroplast dysfunction to signal a retrograde signal and trigger chloroplast degradation [66]. Perhaps an extended period of darkness triggers the accumulation of sugars that cause a similar degradation of pigments in young leaves. The recently emerged leaves of *Arabidopsis* comprise smaller dividing cells containing fewer, smaller-sized chloroplasts undergoing differentiation and biogenesis that could become interrupted by a plastid-derived signal generated during extended darkness. Indeed, chloroplast division/replication can become restricted in spinach leaf discs cultured in the dark or under low intensity green light [53]. Whereas, the enlarged mature cells within older leaves that comprise numerous mature chloroplasts with well-developed thylakoid grana stacks, retain their chlorophylls embedded within the thylakoids and hence remain unaffected by darkness [30, 34, 40, 55]. The biosynthesis and degradation of carotenoids and chlorophylls continuously take place in leaves during light exposed conditions as evident from the carbon isotope labelling with ^14^CO_2_ in *Arabidopsis* [7]. However, dark exposure of pepper leaves downregulated the expression of *PSY* and *PDS* thereby stalling carotenoid biosynthesis [64]. In concert, there was an absence of phytoene accumulation in both young and old *Arabidopsis* leaves from the NFZ treated leaves subject to darkness. Whether darkness and NFZ reduce chlorophyll accumulation and impair chloroplast biogenesis in young leaves by similar signalling mechanisms remains unclear. We propose that darkness blocks the first committed step in carotenoid biosynthesis and/or stalls the supply of isoprenoid substrates from the MEP pathway.

Low temperature affects a broad spectrum of cellular components in plants, including chloroplast development and metabolism [29, 44]. Cold stress can cause irreversible damage to chloroplast structure and photosynthetic capacity and trigger ABA biosynthesis in order to enhance cold acclimation in maize [35]. We showed that colder exposure reduced chlorophyll and carotenoid accumulation in younger leaves. It was previously shown that spinach leaf discs grown at lower temperatures (12 °C continuous) contained small cells and fewer chloroplasts [54]. Cold exposure at 4 °C was also shown to inhibit cell division and arrest growth as evident in *Arabidopsis* roots and maize leaves [3, 60]. Hence, exposure of young leaves to cooler temperatures could suppress cell cycle progression, thereby limiting chloroplast biogenesis and/or division during leaf cell division and/or expansion. The reduction of phytoene and total carotenoid levels in both young and old leaves from the cold exposed plants support an overall reduction in cellular growth processes. This contrasts to higher temperatures that enhanced phytoene accumulation in both young and old leaves yet had no effect on total carotenoid levels and only slightly reduced chlorophyll levels. The reduction in chlorophyll a could be a cellular strategy to reduce heat-induced oxidative stress as was shown in flag leaves at the grain‐filling stage of different heat‐resistant winter wheat varieties [28, 69]. The increased level of phytoene in response to higher temperature could compensate for the higher rate of carotenoid degradation and higher demand of xanthophylls, particularly zeaxanthin which is crucial to maintain functional integrity of chloroplasts [33] and the biosynthesis of apocarotenoids that signal stress events [36]. Low temperature and NFZ exposure have similar effects on chlorophyll and carotenoid biosynthesis in young *Arabidopsis* leaves, contrasting a different signalling mechanism to that induced by darkness which blocks phytoene biosynthesis.

### Chlorophyll levels in young leaves are not altered in mutants that disrupt abscisic acid, strigolactone, and/or aporcarotenoid biosynthesis

Strigolactone and β-apocarotenoid signalling metabolites regulate stress acclimation and plant development [10, 38, 48]. CCD1 cleaves various carotenoid bonds to generate multiple apocarotenoid products [67]. CCD4 cleaves β-carotene [31]. CCD7 catalyses the production of strigolactone from β-carotene [42]. The mature leaves we used to quantify pigment accumulation were unlikely to have transitioned into a phase of senescence and likely harbor mature chloroplasts [34]. It was not surprising that a single *ccd* mutant (*ccd1*, *ccd4*, or *ccd7*) or double mutant combination (*ccd1 ccd4*, *ccd1 ccd7*, *ccd4 ccd7*), were unable to significantly alter chlorophyll or carotenoid levels in older leaves containing mature chloroplasts. The pigment levels reached a threshold in older leaves that remained unchanged in the absence of SL or CCD-derived β-apocarotenoids. The significantly higher level of carotenoid content in the young leaves from *Arabidopsis* single and double mutants indicates that lower carotenoid content in older leaves could not be attributed to CCD catalysed carotenoid degradation. The lower pigment levels of older leaves are more likely due to a combination of lower cell density, less cell division, and a low rate of chloroplast turnover [21, 40, 43, 55]. The recently emerged leaves of *Arabidopsis* comprise numerous smaller and dividing cell types with developing chloroplasts [30, 34] making them amenable to metabolite signals that control chloroplast biogenesis [21, 23]. SLs were shown to positively regulate photosynthesis related genes in tomato [47]. Yet, the chlorophyll levels in younger leaves also remained consistently stable, and higher than older leaves in CCD mutant combinations. In conclusion, it appears that SL or β-apocarotenoid signals are unlikely to regulate chlorophyll accumulation, and hence chloroplast biogenesis in either young emerging or older mature *Arabidopsis* leaves from plants grown under a long photoperiod.

Xanthophylls such as lutein, violaxanthin, and neoxanthin are abundant carotenoids found in photosynthetic leaves, whereas antheraxanthin and zeaxanthin are crucial to maintain the functional integrity of chloroplasts during excessive light or heat stress [22, 61]. Violaxanthin and neoxanthin are precursors of abscisic acid that regulates guard cell closure in stomata, mediates stress acclimation and plant development [25]. Mutants such as *npq2* (ZEP; also known as *aba1-3*, *aba deficient 1*) and *aba4* (NXS) alter xanthophyll accumulation and block ABA biosynthesis in *Arabidopsis* [70]. The consistently higher level of chlorophyll pigments in younger compared to older leaves in *lut2, npq2, lut npq2,* and *aba4 npq1 lut2* mutants, revealed that altering xanthophyll composition and/or their derived oxidation products did not alter chlorophyll levels and therefore may not have affected chloroplast biogenesis. In addition, the lack of ABA biosynthesis in *npq2, lut npq2,* and *aba4 npq1 lut2* mutants revealed that ABA may not regulate chlorophyll levels in young expanding *Arabidopsis* leaves in plants grown under a long photoperiod. Taken together, chlorophyll levels and hence chloroplast biogenesis in young leaves of *Arabidopsis* were not impacted by perturbations in ABA biosynthesis.

### A cis-carotene derived ACS could regulate chloroplast development and chlorophyll accumulation in young leaves

The higher carotenoid and chlorophyll levels in younger compared to older leaves of the *PSY*-OE plants showed that substrate supply was not rate-limiting. The substantially higher level of phytoene accumulation in response to NFZ treated leaves, further supported a continual supply of substrates for carotenoid biosynthesis in young leaves, even when NFZ inhibited chloroplast biogenesis. Therefore, substrate supply into the carotenoid pathway does not necessarily have to be affected for a plastid derived signal to mediate a change in chloroplast biogenesis. *cis*-carotenes have been recently proposed to act as substrates in generating an ACS that controls plastid development (e.g. etioplast, chromoplast, and chloroplast) [5, 14, 26]. Phytoene and phytofluene accumulation in response to NFZ treatment are unlikely to be signals themselves, although a burst in their production was shown to elicit artificial chloroplast-to-chromoplast differentiation in leaves [45]. Etiolated cotyledons of *z-iso* accumulate *cis*-carotenes; 15-*cis*-phytoene, 15, 9’ di-*cis*-phytofluene, and 9,15,9’-tri-*cis*-zeta-carotene [14]. The *z-iso* mutant was shown to generate a subtle yellow leaf phenotype when plants were grown under a shorter photoperiod, and there was a slight effect on plastid development as de-etiolated seedlings showed a subtle reduction in chlorophyll accumulation in cotyledons after light exposure [14]. However, the younger leaves from *z-iso* mutants grown under a longer photoperiod displayed chlorophyll levels similar to that of WT. Therefore, *cis*-carotenes that accumulate when isomerisation is impaired by *z-iso* are not provide the right precursors for the biosynthesis of a *cis*-ACS that regulates chlorophyll accumulation and plastid development in photosynthetic tissues.

ζ-carotene, neurosporene and/or tetra-*cis*-lycopene have been direct linked to the generation of a yet to be identified *cis*-ACS in *crtiso* mutant tissues that regulate PLB formation in etioplasts and chloroplast biogenesis in plants grown under a shorter photoperiod causing young leaves to display a virescent phenotype [14, 50]. Sufficient light exposure facilitates photoisomerisation of tetra-*cis*-lycopene into all-*trans*-lycopene, thereby reducing *cis*-carotene accumulation, restoring plastid development, and greening in older leaves of the *Arabidopsis crtiso* as well as tomato *tangerine* mutants [14, 39]. Indeed, we observed similar levels of chlorophyll in older leaves of the *crtiso* mutant relative to WT plants growing under a longer photoperiod. Surprisingly, the level of total chlorophyll, lutein, and β-carotene were similar between young and old leaves of *crtiso* when plants are grown under a longer photoperiod, revealing that a *cis*-carotene derived ACS could be produced under longer photoperiods in order to control plastid development. However, the levels of violaxanthin, zeaxanthin and antheraxanthin were considerably higher in younger relative to older leaves revealing that such a *cis*-ACS regulates chlorophyll pigmentation and individual carotenoid biosynthesis by different mechanisms. We conclude that isomerisation mediated by CRTISO, and perhaps photoisomerisation, are major rate-limiting steps in regulating a plastid-derived signal that controls chloroplast development during cell division and expansion in young emerging leaves.

Given there were similarities (e.g. reduced chlorophyll accumulation) and differences (differential carotenoid biosynthesis) in how *crtiso* and NFZ impact upon pigment levels in young leaves, this prompted us to explore if they perturb similar signalling pathways. We previously proposed that the *crtiso*-mediated *cis*-ACS regulated gene expression was independent of *genomes uncoupled* (*gun*)-mediated NFZ retrograde signalling [14]. Here, we demonstrated that NFZ heightened the accumulation of phytoene in *crtiso* revealing that neither NFZ nor *crtiso,* or both in combination, impaired substrate supply for carotenogenesis. However, NFZ should have blocked the production of the downstream *cis*-ACS, as it was able to restore PLB formation in *crtiso* etiolated seedlings [19]. The treatment of *crtiso* with NFZ further decreased chlorophylls, lutein and total carotenoids below that of NFZ treated WT older leaves, and collectively reduced violaxanthin and antheraxanthin to levels below that of *crtiso* older leaves. This additive effect indicated that NFZ and *crtiso* generate different signals, neither of which affected the level of the stress pigment zeaxanthin that remained higher in younger leaves, irrespective of the treatment or genotype. It seems likely that the *cis*-ACS produced by *crtiso* and retrograde signal (e.g. Mg-ProtoIX) generated by NFZ inhibition of PDS activity, act in different manners to control chloroplast development and maintain pigment homeostasis in young leaves.

## Conclusion

The *Arabidopsis* foliar pigment-based signalling bioassay has utility to combine chemical (e.g. NFZ), environmental (darkness and temperature), and genetic (e.g. *crtiso* or *gun* signalling mutants) perturbations to decipher how plastid-generated signalling metabolites operate mechanistically *in planta* to control chlorophyll biosynthesis and chloroplast development. NFZ treatment of whole rosettes likely triggered a chlorophyll-derived signal that affected chloroplast biogenesis during early leaf development leading to a reduction in chlorophyll levels. The *Arabidopsis* foliar pigment-based signalling bioassay allowed us to demonstrate that carotenoid isomerization was the key rate-limiting step in the carotenoid pathway controlling chlorophyll biosynthesis in leaves from plants grown under a longer photoperiod. The *Arabidopsis* foliar pigment-based signalling bioassay further evidences that impaired carotenoid isomerisation triggers the production of a signal that is likely to control chloroplast biogenesis.

## Methods and materials

### Plant material and growth conditions

This study was performed using wild type (WT) and a wide array of mutant and transgenic lines of *Arabidopsis thaliana* (ecotype Col-0) including *ziso* (*z-iso*) [18], *crtiso (ccr2.1)* [50], *lut2* [51], *ccd1(ccd1-1)*, *ccd4* (SALK097984C) and *ccd7 (max3-11)* [13], 35S::At*CRTISO* [16], 35S::AtPSY#23[46], *npq2, lut2 npq2,* and *aba4 npq1 lut2* [70], and *ccd1 ccd4*, *ccd1 ccd7,* and *ccd4 ccd7* [57].

*Arabidopsis* plants were grown in growth cabinets (Climatron Star700, Thermoline Scientific, Australia) or walk-in room equipped with the controlled plant growth conditions. Debco Seed Raising and Superior germinating mix (Scotts Australia) was supplemented with 3% Osmocote slow-releasing fertiliser (Garden City Plastics Australia) and used to grow plants in 30-Cell Kwikpot trays (Cell dimensions: 50 × 50 × 60 mm, Cell volume: 100 ml, Garden City Plastics, Australia). *Arabidopsis* seeds were sown on the moistened soil mix and stratified for three days at 4 °C in the dark. Individual plants were grown under a 16/8 h light/dark photoperiod illuminated by cool fluorescent lamps (130–150 µ mol m^−2^ s^−1^) at 22/18 °C (day/night) temperature cycle, unless otherwise stated.

*Arabidopsis* rosette leaves at different developmental stages were collected (20–50 mg/sample) 7 to 9 h after illumination, snap-frozen in liquid nitrogen, and stored in − 80 °C prior to quantify pigment levels. The ontogeny-based numbering of rosette leaves was numbered serially as described [12, 32].

### Pigment-based signalling bioassay for Arabidopsis leaves

The three-week-old soil-grown *Arabidopsis* plants were used in a pigment-based retrograde signal bioassay developed *in-planta*. Plants were kept in the dark for four hours prior to experimentation to establish a metabolic equilibrium. Dark-adapted rosettes were detached from the rootstock keeping a 5 mm portion of the hypocotyl intact and transferred onto a Kimwipe paper towel within a plastic Petri dish saturated with 10 ml of NF (50 μM; or as indicated) solution or MilliQ. Three to four plants (10 to 15 leaves) were incubated per petri dish, covered with a clear plastic lid, and incubated (24 h: or as indicated) under the continuous light or darkness at 22 °C. The mature fully expanded (leaves 1 to 4; old) and recently emerged (leaves 9 to 13, young) leaves (Fig. 1B) were collected after 24 h of treatment and stored in -80 °C prior to quantifying pigments. For the dark incubation experiments, leaf tissues were harvested under a green LED light.

### Pigment extraction and quantification

Pigment extraction, quantification and analysis was performed as previously described [22, 23]. In brief, frozen tissues were milled in TissueLyser® (QIAGEN; 2 min, 20 Hz) using stainless steel beads (~ 3 mm diameter) until finely powdered. Pigments were extracted in 1 ml of acetone and ethyl acetate (60:40 v/v) containing 0.1% (w/v) butylated hydroxytoluene. The mixture was vortexed, centrifuged (15,000 rpm for 5 min at 4 °C) and the upper ethyl acetate phase analysed using a HPLC (Agilent 1260 Infinity) equipped with YMC-C30 (250 × 4.6 mm, S-5 μm) column and Diode Array Detector (DAD) detector. A 35-min reverse phase method was used to separate carotenoids. This consisted of a 5 min isocratic run of 100% solvent A (methanol: triethylamine, 1000:1 v/v) followed by 20 min ramp to 100% solvent B (methyl *tert*-butyl ether) and 2 min isocratic run of 100% solvent B with a solvent flow rate of 1 ml/min. Carotenoids and chlorophylls were identified based upon retention time relative to known standards and their light emission absorbance spectra at 440 nm (chlorophyll, β-carotene, xanthophylls), 340 nm (phytofluene) and 286 nm (phytoene). Absolute quantification and determination of composition of pigments was performed as described [1, 2, 14]. Quantification of phytoene and phytofluene was expressed peak area per milligram (mg) fresh weight.

### Data analysis

One- or Two-Way ANOVA was performed using the Holm-Sidak *post-hoc* multiple comparisons to determine significant interactions within, and across, the test groups in response to the various treatment conditions.

## Data Availability

The data that support the findings of this study are available from the corresponding author upon reasonable request.
